# Three-Dimensional (3D) Printing of Polymer-Metal Hybrid Materials by Fused Deposition Modeling

**DOI:** 10.3390/ma10101199

**Published:** 2017-10-19

**Authors:** Susanna Fafenrot, Nils Grimmelsmann, Martin Wortmann, Andrea Ehrmann

**Affiliations:** Faculty of Engineering and Mathematics, Bielefeld University of Applied Sciences, 33619 Bielefeld, Germany; susanna.fafenrot@fh-bielefeld.de (S.F.); nils.grimmelsmann@fh-bielefeld.de (N.G.); martin.wortmann@fh-bielefeld.de (M.W.)

**Keywords:** 3D printing, FDM technology, hybrid printing materials, metal filament, tensile strength, flexural modulus

## Abstract

Fused deposition modeling (FDM) is a three-dimensional (3D) printing technology that is usually performed with polymers that are molten in a printer nozzle and placed line by line on the printing bed or the previous layer, respectively. Nowadays, hybrid materials combining polymers with functional materials are also commercially available. Especially combinations of polymers with metal particles result in printed objects with interesting optical and mechanical properties. The mechanical properties of objects printed with two of these metal-polymer blends were compared to common poly (lactide acid) (PLA) printed objects. Tensile tests and bending tests show that hybrid materials mostly containing bronze have significantly reduced mechanical properties. Tensile strengths of the 3D-printed objects were unexpectedly nearly identical with those of the original filaments, indicating sufficient quality of the printing process. Our investigations show that while FDM printing allows for producing objects with mechanical properties similar to the original materials, metal-polymer blends cannot be used for the rapid manufacturing of objects necessitating mechanical strength.

## 1. Introduction

Three-dimensional (3D) printing belongs to the emerging topics of our time. On the one hand enabling localization and individualization of production [1], it offers on the other hand new opportunities to produce objects that would have been difficult or even impossible with former technologies [2].

Different technologies enable 3D printing, such as stereo-lithography, selective laser sintering, or fused deposition modeling (FDM) [3]. Especially if the FDM technology is of high technological interest since most inexpensive printers are based on this principle. Here, a polymer filament is guided through a heated nozzle where the material is molten and deposited at defined positions on a printing bed. After finishing the first layer, the distance between printing bed and extruder nozzle is increased, and the second layer is printed on the first one, etc. [4].

In FDM printing, usually diverse thermoplastic polymers are used, such as poly (lactic acid) (PLA), acrylonitrile butadiene styrene (ABS), polyamide 6.6 (PA 6.6), polycarbonate, etc. [5]. Additionally, several special filaments exist, combining polymers with different other materials, such as wood, brick dust, or metal particles. These filaments, however, are in the moment only used for printing models, not objects that may be stressed mechanically, since their mechanical properties are known to be reduced in comparison with pure polymer filaments. Nevertheless, scientific examinations of the influence of the printing parameters on the mechanical properties of these special filaments are scarce.

Polymer-metal blends have already been used for FDM printing of metal-oxide semiconductors using a sintering step after 3D printing [6]. Polymers loaded with metal oxide nanoparticles, such as TiO_2_ or MoO_3_, were FDM printed and tested with respect to their antimicrobial and antifungal properties [7]. 3D printing with electro-conductive materials was tested by diverse groups, using techniques from laser-assisted writing [8] and metallic inks [9,10] to filling conductive liquids into 3D printed substrates [11,12] to direct FDM printing [13].

In spite of these inspiring research approaches giving rise to novel technological applications of the FDM process, only one group at this time published research describing the influence of filling ABS with different amounts of metal particles and printing such a hybrid filament at different temperatures and with different infill densities, indicating that increased temperatures result in increased tensile stress and elongation at break [14,15].

Our article thus describes the mechanical properties of two commercially available metal-polymer hybrid filaments, based on PLA, influenced by different printing parameters, and measured in elongation and bending tests. PLA was chosen as the base material due to several reasons: Firstly, it belongs-together with ABS-to the most often used polymers in FDM printing. PLA is of special interest due to its biocompatible, biodegradable, non-toxic, non-immunogenic, and non-inflammatory properties, making the material usable in medical applications [16]. In biomedical applications, degradation is often advantageous, especially due to the lack of toxicological risks [17]. Additionally, PLA offers the best adhesion on textile materials and is thus also suited to add new mechanical properties or design aspects to technical textiles or garments [18,19]. The latter aspect is of special importance for metal-filled printing polymer, which can be expected to show a reduced mechanical strength, but interesting optical properties, suggesting their use in combination with another material with higher tensile strength. Finally, PLA is known to show shape-memory properties, such as self-repairing processes or self-fitting of implants [20,21,22,23,24,25,26], making it useful in diverse applications where a shape change due to a temperature change is desired.

## 2. Results

First, TGA (thermo-gravimetric analysis) measurements were performed to examine the metal fractions of the hybrid filaments under investigation, resulting in 78 wt % bronze in the Bronzefill filament and 46 wt % iron in the Magnetic Iron filament, respectively. These values correspond—for average densities of 8150 kg/m³ (bronze), 7860 kg/m³ (iron), and 1320 kg/m³ (PLA)—to approximately 36 vol % of metal in Bronzefill and 12 vol % of metal in Magnetic Iron, respectively.

**Figure 1:**
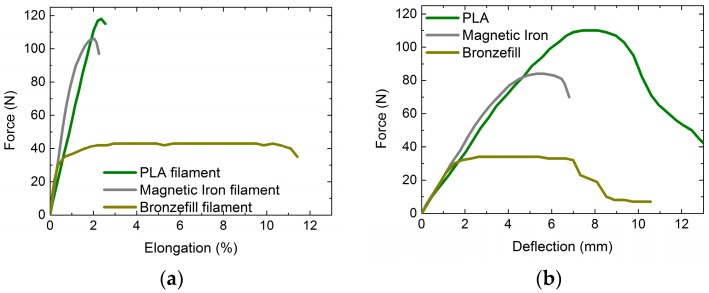
Typical examples of force-displacement curves: (**a**) Force-elongation measurements of the three filaments used in this study; (**b**) Force-deflection curve measured for bending specimens printed from the three filaments used here.

Figure 1 shows the results of tensile and bending tests, performed on the different filaments (Figure 1a) and some bending specimens (Figure 1b), respectively. All of the measurements were performed between 1 and 2 days after 3D printing; however, tests with all three materials approximately 15 min after printing (when the specimens were just cooled down) revealed no difference in tensile and bending tests. PLA and Magnetic Iron filaments show similar force-elongation curves, with PLA having a higher elongation at break and a higher tensile strength. The Bronzefill filament, however, starts elongating plastically already at low forces and is stretched by more than 10% until it finally breaks.

Similar behavior is visible in the bending tests. Here, PLA again shows a higher force and deflection at break than Magnetic Iron, with the PLA specimen not breaking for the standard conditions depicted here (cf. Table 1). Similar to the tensile test, the Bronzefill specimen starts plastic deformation for small forces and is deflected plastically for several millimeters until first (incomplete) breaks occur.

From this first test, it can already be stated that the Bronzefill filament with a high metal filling shows mechanical properties significantly different from pure PLA or Magnetic Iron filament which contains less metal particles.

In the following figures, the tensile strengths and flexural strength of specimens produced from the three materials are depicted. Both bending and elongation samples were produced with three different infill degrees, i.e., 20%, 60%, and 100%. Opposite to common casting methods known for plastics or metals, this light weight construction method is typical for 3D printing. This means that the cross-section of the samples is not always completely filled and that calculations of tensile and flexural strengths must be modified or, alternatively, interpreted correctly.

For the tensile strength, the average cross-section of the samples was calculated by taking into account the numbers of completely filled outer layers and the infill degree, i.e., the completely filled cross-section was only reached for 100% infill degree, while lower infill degrees resulted in smaller nominal cross-sections. These calculated values were verified by comparing the measured masses of all samples, comparing lower infill degrees to samples with 100% filling.

For the flexural strengths, scaling the dimensions of the bending specimens in a similar way is not possible since not only the amount of material in the cross-section is necessary for its calculation, but also the material’s distribution, influencing the moment of inertia. Thus, the values of the flexural strength are always depicted with respect to the outer dimensions of the bending specimens.

This means that the values of the tensile strength depicted here should be identical for all of the infill degrees, while the values of the flexural strength must be larger for higher infill degrees.

**Figure 2:**
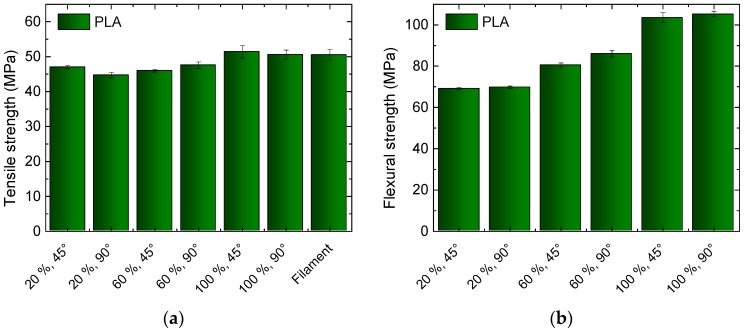
Results of tests on common poly (lactide acid) (PLA), three-dimensional (3D) printed with different infill degrees and orientations: (**a**) Tensile strengths for the different specimens and the original filament; (**b**) Flexural strengths for the different specimens.

Figure 2 depicts the tensile strength and flexural strength, measured for PLA, using different infill degrees and orientations. The infill pattern is “rectilinear”, i.e., even lines are printed in the not completely filled area (cf. Table 1), oriented rectangularly in consecutive layers, with the angles between printed lines and the perimeter being either +45°/−45° or 90°/0°.

For the tensile strength, both of the sets of samples with 100% infill degree reach approximately the value of the original filament, which can be assumed to be the maximum possible value. This means that during the 3D printing process, the same adhesion between the neighboring printed lines within one layer are reached as in the filament extrusion process. For lower infill values, the tensile strengths are reduced, which can be explained by the open parts in the inner layers that prevent the intra-layer adhesion. Instead, only lines printed in consecutive layers adhere to each other in small areas.

For the flexural strength, a typical lightweight construction effect becomes visible—the samples printed with 60% infill degree show significantly more than 60% of the maximum flexural strength reached with 100% infill degree. The difference between 20% and 60% is even smaller.

It should be mentioned that while the flexural strength is mostly similar to or slightly higher than the tensile strength of a specimen, it is not unusual to find values of the flexural strength significantly higher than the tensile strength, e.g., for 3D printed PLA objects with and without carbon fiber reinforcement [27].

**Figure 3:**
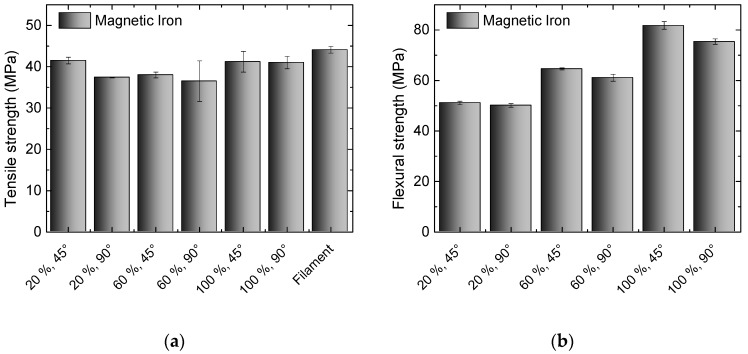
Results of tests on Magnetic Iron, 3D printed with different infill degrees and orientations: (**a**) Tensile strengths for the different specimens and the original filament; (**b**) Flexural strengths for the different specimens.

While PLA is only depicted here as a benchmark, the Magnetic Iron filament already contains an amount of metal that influences the mechanical properties as well as the masses of these samples. While the masses are increased, as compared to PLA samples, by a factor of approximately 1.58, the mechanical properties are depicted in Figure 3. Here, the tensile strength of the filament is not completely reached by the 3D printed test specimens, indicating that the adhesion within one printed layer is not perfect and can probably be marginally increased by modifications of the printing parameters. This results in a slightly reduced tensile strength, when compared to pure PLA.

In the flexural strength, opposite to pure PLA, differences between both infill orientations become visible, with the 45° orientation always showing slightly higher values. Additionally, opposite to PLA, all of the samples broke during the test, while PLA samples only showed stress whitening. The values reached here are significantly reduced in comparison with pure PLA, indicating that the inter-layer adhesion is also reduced, resulting in smaller forces necessary to deflect the samples.

**Figure 4:**
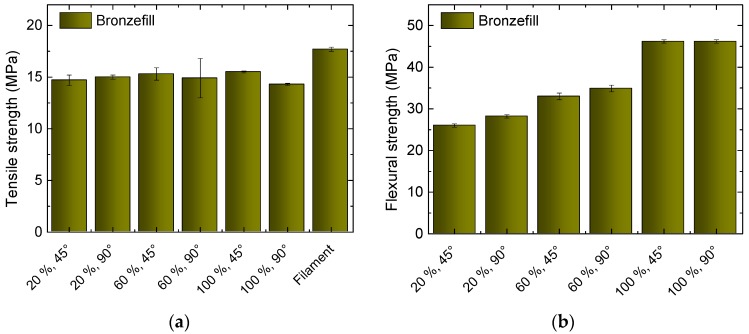
Results of tests on Bronzefill, 3D printed with different infill degrees and orientations: (**a**) Tensile strengths for the different specimens and the original filament; (**b**) Flexural strengths for the different specimens.

Next, Figure 4 depicts the results of the Bronzefill measurements. Here again, the value of the filament is not reached in the tensile tests of the 3D printed specimens, nor do the samples with 100% show infill a higher tensile strength than those with reduced infill degrees.

In the flexural strength, the ratio of the values at different infill degrees is similar to those measured for the other two filaments, here not showing any significant difference between 45° and 90° orientation. Bronzefill samples never broke in bending tests.

Generally, tensile and flexural strengths are significantly reduced, when compared to pure PLA or even Magnetic Iron, by a factor of 2–3. These factors are higher than expected due to the amount of approximately one third of the polymer, which is exchanged by metal. However, this can be explained by the missing cohesive bonds inside the PLA matrix due to embedment of metal particles.

For Magnetic Iron, the discrepancy between the percentaged loss of tensile and flexural strength is also higher than the approximately 12% metal content, but the effect is less severe than for Bronzefill.

**Figure 5:**
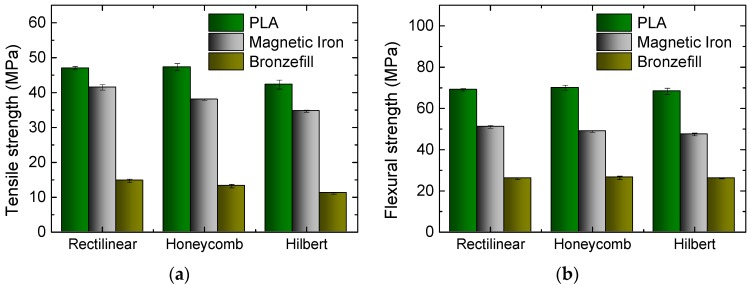
Results of tests on the three materials under examination, 3D printed with different infill patterns: (**a**) Tensile strengths for the different specimens; (**b**) Flexural strengths for the different specimens.

In the next test, different infill patterns are compared for samples printed with 20% infill degree and 45° infill orientation. Figure 5 depicts the results. Unexpectedly, the rectilinear pattern—which is printed fastest-results in the highest tensile strength for all materials. Honeycomb (cf. Table 1)—which is often regarded as the most stable one—shows slightly reduced values, followed by Hilbert, which is known to be relatively unstable. The latter is built like a labyrinth with 90° corners after a few millimeters, identical for all of the layers (cf. Table 1). Both honeycomb and Hilbert patterns do not include straight lines connecting the outer shells, opposite to the rectilinear pattern, which is considered to be the reason for their reduced tensile strengths.

A different result is found in the flexural strength. Here, the three infill patterns give nearly identical results. The honeycomb pattern—which is often used in lightweight construction—is never significantly advantageous in comparison to rectilinear and Hilbert filling patterns.

Apparently, the choice of the infill pattern has to be based on the planned application, with the rectilinear pattern having better tensile properties, while under bending all of the patterns show similar strength.

In further experiments, different printing temperatures were tested, with temperatures of 200 °C/210 °C/220 °C chosen for PLA and Magnetic Iron, while Bronzefill was printed with 220 °C/230 °C/240 °C, since 220 °C was the lowest temperature at which it could be printed reliably.

**Figure 6:**
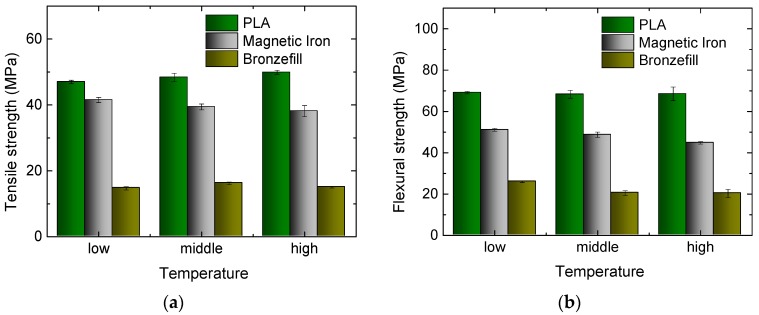
Results of tests on the three materials under examination, 3D printed at different nozzle temperatures: (**a**) Tensile strengths for the different specimens; (**b**) Flexural strengths for the different specimens.

Opposite to the findings described in References [14,15], no significant influence of the nozzle temperature is visible in Figure 6; only a slight increase of PLA tensile strength and a slight decrease of Magnetic Iron and Bronzefill flexural strength with increasing printing temperature can be recognized.

**Figure 7:**
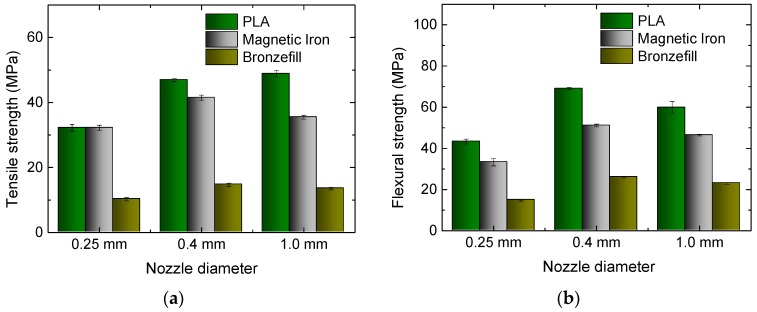
Results of tests on the three materials under examination, 3D printed with different nozzle diameters: (**a**) Tensile strengths for the different specimens; (**b**) Flexural strengths for the different specimens.

Finally, the influence of the nozzle diameter was examined. Besides the usual nozzle diameter of 0.4 mm and the also commercially available diameter of 0.25 mm, a nozzle was modified to have a diameter of 1.0 mm. Figure 7 depicts the results. For the smallest nozzle diameter, the tensile strengths of all of the materials are lower than for the 0.4 mm nozzle. Unexpectedly, PLA and Magnetic Iron show identical values for both tensile and flexural strength. This may be explained by the high printing quality of Magnetic Iron, without any deviations from the desired infill pattern, while for PLA, small printing errors were visible during the infill printing process.

For the largest nozzle, PLA shows slightly higher tensile strength than for the usual diameter, while the other filaments perform best with the middle nozzle. This may be explained by the pure PLA necessitating lower temperatures to flow regularly, combined with a temperature gradient in the unusually large nozzle, resulting in potentially too low temperatures in the middle of the nozzle, which may be more problematic for the hybrid materials. On the other hand, the smallest nozzle will probably produce a less regular material flow due to sporadic choking.

In the flexural strength, the standard nozzle diameter of 0.4 mm results in the best values, as can be expected from the arguments above.

**Figure 8:**
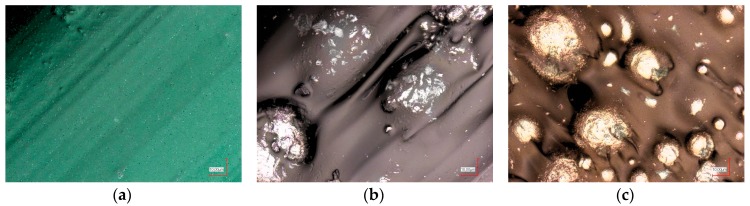
Confocal laser scanning microscope (CLSM) images of (**a**) PLA; (**b**) Magnetic Iron; and (**c**) Bronzefill specimens after 3D printing.

To understand these effects on a microscopic scale, microscopic and confocal laser scanning microscope (CLSM) images were taken. The CLSM images depicted in Figure 8 show that PLA has a smooth, even, and homogeneous surface structure. In Magnetic Iron, the metal particles are clearly visible at the sample surface. The bronze particles included in the Bronzefill filament seem to have a broader diameter distribution; the black area near the middle of Figure 8c shows a region where probably a bronze sphere is already abraded.

**Figure 9:**
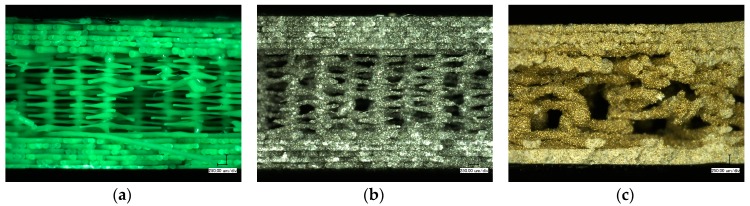
Microscopic images of specimens after tensile tests, prepared from (**a**) PLA; (**b**) Magnetic Iron; and (**c**) Bronzefill.

In Figure 9, the cross-sections of specimens after tensile tests are visible. For PLA and Magnetic Iron, all of the broken lines of the infill area show a diminution next to the break, which is not visible in the completely filled top and bottom areas. Apparently, failure starts with a visible elongation in the infill area, while the lines in top and bottom areas are too strongly connected for being elongated and at the same time reduced along their radii. While Magnetic Iron already shows a higher irregularity of the infill lines, as compared to PLA, this effect is further increased for Bronzefill. Additionally, the distribution of the bronze particles is quite inhomogeneous. Such inhomogeneities will result in some areas being less strong—i.e., containing less polymer material and more bronze—than others, becoming the “weak links” in the whole sample. This explains the small tensile and flexural strengths measured for all Bronzefill samples.

**Figure 10:**
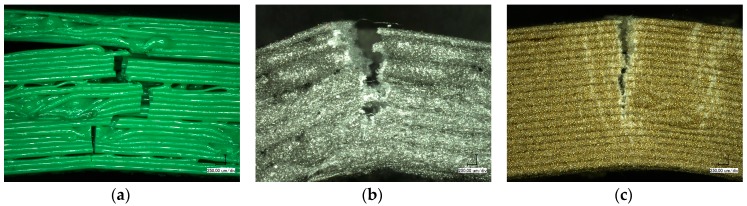
Microscopic images of specimens after bending tests, prepared from (**a**) PLA; (**b**) Magnetic Iron; and (**c**) Bronzefill.

Figure 10 depicts digital microscope images of bending specimens. For PLA, failure occurs via a superposition of delamination between different layers and breaks of layer bundles. According to Figure 9a, this can be explained by the relatively small contact areas and thus weak links between consecutive layers especially in the infill area.

For Magnetic Iron, no sample showed delamination; instead one progressing break line is visible. Along the line, stress whitening can be recognized. This can be understood since in Figure 9b, the contact areas between neighboring infill lines seem to be larger due to the uneven, “smeared” geometry of the single lines, reducing the danger of delamination. At the same time, the mechanical strength of each line is reduced due to the included metal particles. Both effects, taken together, result in a modified failure mechanism when compared to PLA.

Bronzefill, finally, shows several stress whitening lines surrounding the progressing break line. The large amount of metal in this material leads to a high elongation at break (Figure 1a), which results in Bronzefill specimens never breaking during bending tests.

In addition to the tensile strength and the flexural strength, the measurements performed in this study also allow for calculating the elastic modulus and the flexural modulus. However, as it could already be assumed from Figure 1, these values describing the elastic deformation of the specimens are quite similar for all of the materials and are nearly identical for the specimens produced with the same material using different printing parameters. The elastic and the flexural modulus are only significantly reduced for all of the samples printed with the small nozzle, which is similar to the finding that tensile and flexural strength are significantly reduced in this case, too.

**Figure 11:**
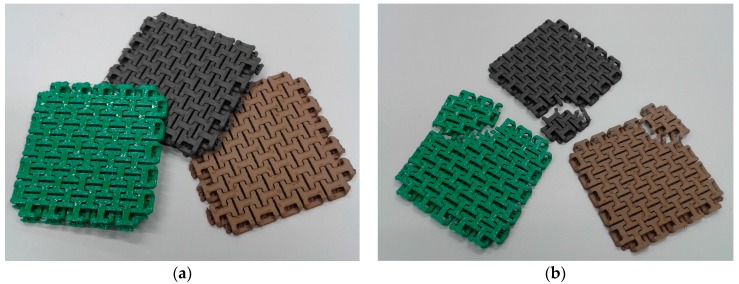
Chain mail, 3D printed from different filaments (green: PLA; black: Magnetic Iron; brown: Bronzefill): (**a**) Printing results; (**b**) Results of tensile tests. Lateral dimensions of the three samples are 80 mm.

Instead, chain mail structures were printed with the three filaments, as depicted in Figure 11a. These structures are slightly flexible due to the interconnected chain links. They were printed without the support structure. While the pure PLA chain mail structure (green) could easily be untightened by carefully breaking the undesired connections between the single chain links, this process was harder and needed more time for the Bronzefill sample since the latter material tends to “smearing” and forming undesired additional connections.

The tensile tests resulted in broken sub-structures at low forces (Figure 11b); this approach did not lead to the desired force distribution between large numbers of small sub-structures. As visible in Figure 11b, failure happened along the thinnest parts of the single chain links. Instead of making the structure stronger by creating a larger area, the weak links in this structure break easily, as compared with the compact tensile and bending test samples.

## 3. Discussion

Our experimental results give rise to the influence of different material and printing parameters on the mechanical properties of polymer-metal hybrid FDM-printed objects.

When comparing pure PLA with the PLA blends Magnetic Iron (46% metal) and Bronzefill (78% metal), the results revealed higher tensile and flexural strengths than expected due to the polymer fractions of these materials.

No significant influence could be measured for the filling pattern or the printing temperature. The nozzle diameter, on the other hand, clearly influenced the mechanical properties of the specimens, with the custom nozzle diameter of 0.4 mm being ideal in most cases.

Comparing the tensile strengths of the 3D printed materials with the values of the pure filaments showed for PLA and Magnetic Iron nearly no difference, indicating that the 3D printing process results in ideal inter- and intra-layer adhesion. For Bronzefill, a significant difference between the values of the filament and the 3D printed objects is visible, showing that for this filament the printing process can still be optimized.

Especially for Bronzefill with its relatively high amount of metal particles, the interfacial adhesion between PLA and metal plays an important role. While the influence of printing parameters on the mechanical properties of the resulting specimens has been studied for pure polymers [28,29], no reports about the mechanical properties of metal-polymer samples were found in the literature. Nevertheless, PLA is sometimes blended with silver nanowires to increase conductivity [30] or metal-organic frameworks to increase also the mechanical properties of the resulting nanocomposites [31]. This shows that including significantly smaller metal particles, most probably in the form of nanofibers, may even support the mechanical properties of 3D printed objects. While nowadays commercially available FDM polymers are either blended with almost round metal particles or carbon fibers/graphene sheets, combining metallic properties with fiber shapes may introduce new mechanical and also electrical properties.

Here, however, the interfacial adhesion is apparently significantly reduced in comparison with the adhesion inside the pure polymer. This can be recognized from Figure 9c. Here, the base layers show a significantly different color than the other layers along the break. Keeping in mind that the average ratio of metal in Bronzefill is 36 vol %, this ratio corresponds approximately to the visible amount of metal particles in the lower layers of the Bronzefill sample. In all of the upper layers, however, the fracture plane seems to consist nearly purely of metal. This can be explained as follows: while TGA measurements have revealed that the metal fraction in the filament changes only by approximately 2 wt %, comparing different areas or the filament, on smaller scales variations of the metal content occur during printing. In the lower layers, which are completely filled, these metal agglomerations are not critical. In the upper layers, especially in the relatively open infill area with only few contacts between the printed lines, each line can break at the weakest point, which is where most metal is agglomerated.

This effect also explains why only for Bronzefill there is a significant different between the tensile strengths of the filament and the printed samples. Apparently for Bronzefill, another printing strategy has to be chosen as for pure PLA, i.e., printing should be performed creating as densely filled specimens as possible to avoid disproportionately high decrease of the mechanical properties with an increase of the metal content. On the other hand, increasing the metal-polymer interface adhesion would significantly increase the mechanical properties of such hybrid printing material and should thus be investigated in the future.

**Figure 12:**
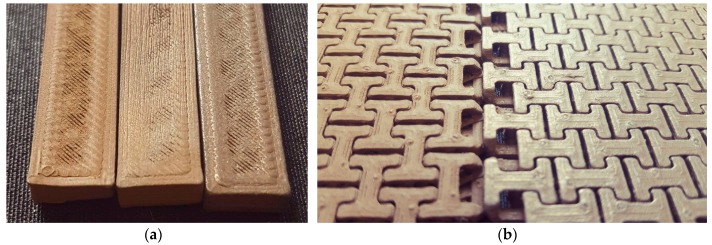
Example of Bronzefill samples: (**a**) Bending test specimens without polishing (**left**), polished with a steel brush (**middle**) and by inserting the specimen in a rotating drum filled with small screws (**right**), sample widths of 10 mm; (**b**) Chainmail (for an overview image, cf. Figure 11) before (**left sample**) and after polishing in a rotating drum (**right sample**), chain link width of 12 mm.

Generally, while FDM printing of polymer-metal hybrid materials can not be expected to result in objects as strong as pure polymers or even metals, this study shows that small metal concentrations can be embedded into a polymer filament, resulting in magnetic, conductive, optic, or other desired properties, without severely decreasing the tensile and flexural properties. As an example, Figure 12 shows the effects that can be created with the Bronzefill sample by polishing it in different manners. Tests were performed on bending test specimens (Figure 12a) and chain mail samples (Figure 12b). The first ones were polished using a steel brush, showing a slight increase of gloss (middle specimen in Figure 12a) as compared to the original sample (left specimen in Figure 12a). Additionally, a bending specimen was polished by inserting it into a rotating drum filled with small screws; this resulted in higher gloss (right specimen in Figure 12a). The chain mail samples were damaged by the steel brush and thus only polished with the rotating drum. Figure 12b shows the difference between the original sample (left specimen) and the polished one (right specimen). Here, the increased gloss due to the polishing process is clearly visible.

Future research should concentrate on optimizing the polymer-metal interface adhesion, modifying the dimensions and shapes of the integrated metal particles, and testing material combinations of polymers with high and metals with low melting points, possibly allowing for extending the recent polymer extrusion to a polymer-metal co-extrusion technology.

Additionally, due to the possibility to combine PLA based FDM polymers with textile materials, combining filaments with high amounts of metal should be tested with respect to possible stabilization (i.e., increase of tensile and flexural strength) of the samples. Especially in combination with the shape-memory properties of PLA, this might allow for using metal-polymer hybrid materials in applications with higher mechanical stress.

## 4. Materials and Methods 

For 3D printing, the FDM printer Orcabot XXL (Prodim, Helmond, The Netherlands) was used. The printing parameters are depicted in Table 1, with the standard parameters (which were used if not mentioned otherwise) marked with light-grey background.

The filament materials used here were PLA, Magnetic Iron (Proto-pasta, Vancouver, WA, USA), and Bronzefill (Colorfabb, Belfeld, The Netherlands), all were purchased from Filamentworld, Neu-Ulm/Germany.

Since the Bronzefill filament could not be printed using temperatures below 220 °C, here the temperature dependence was examined using higher temperatures.

The layer thickness was chosen as half the nozzle diameter, i.e., layers printed with 0.25 mm nozzle diameter had a thickness of 0.125 mm, etc. The width of each printed line corresponded with the nozzle diameter.

The thickness of the complete top and bottom layers was left nearly equal for all three of the nozzle diameters, i.e., four layers with 0.125 mm height each for the 0.25 mm nozzle, two layers with 0.2 mm height for the 0.4 mm nozzle, one layer with 0.5 mm height for the 1 mm nozzle. Similarly, the numbers of perimeters were set to the value of approximately 0.8 mm for all three nozzles.

Measurements of the tensile strength and the flexural modulus were performed using a Sauter universal testing instrument. Dimensions and measurement parameters of the tensile and bending specimens were defined according to EN ISO 527-1:2012 (tensile strength) and ISO 178 (flexural strength). The stl file for printing the specimens for the tensile tests was created by Piotr Cichalewski [33], the stl file for printing the chain mail was generated by Itai Nahshon [34].

TGA measurements were performed on a Hi-Res TGA 2950 Thermogravimetric Analyzer (TA Instruments, New Castle, DE, USA). Samples of approximately 25 mg were cut from the untreated filaments. The samples were then heated to 450 °C in nitrogen and to 500 °C in synthetic air at a constant heating rate of 30 °C/min. The remaining mass of the sample corresponds to the metal fraction of the hybrid filament.

Images of sample surfaces and fracture areas were taken using a digital microscope VHX-600D with a nominal magnification of 50× and a confocal laser scanning microscope (CLSM) VK-9000 with a nominal magnification of 2000×. Both microscopes are from Keyence, Neu-Isenburg, Germany.

## Figures and Tables

**Table 1:** Printing parameters used in this study. Marked values were used as standard values. Infill patterns from [32].

Parameter	Value 1	Value 2	Value 3
Nozzle diameter	0.25 mm	0.4 mm	1 mm
Infill degree	20%	60%	100%
Infill orientation	45°	-	90°
Infill pattern	Rectilinear	Honeycomb	Hilbert curve
Sketch of infill pattern	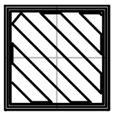	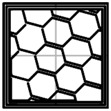	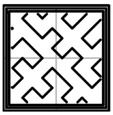
Printing temperature (PLA, Magnetic Iron)	200 °C	210 °C	220 °C
Printing temperature (Bronzefill)	220 °C	230 °C	240 °C
Printing bed temperature	-	60 °C	-
Thickness of complete layers (top/bottom)	-	0.45–0.5 mm	-
Thickness of complete perimeters	-	0.8–1.0 mm	-
